# Serum copper assessment in patients with polycystic ovary syndrome and tubal infertility: A retrospective 5‐year study

**DOI:** 10.1002/fsn3.4258

**Published:** 2024-06-10

**Authors:** Yanping Liu, Wei Zhang, Zhenxing Liu, Aiyan Zheng, Baoquan Liang, Hong Li, Qingxia Meng

**Affiliations:** ^1^ Center of Reproduction and Genetics The Affiliated Suzhou Hospital of Nanjing Medical University, Suzhou Municipal Hospital, Gusu School of Nanjing Medical University Suzhou China; ^2^ Department of Obstetrics and Gynecology The Affiliated Suzhou Hospital of Nanjing Medical University, Suzhou Municipal Hospital Suzhou China

**Keywords:** infertility, IVF cycle characteristics, lipids metabolism, polycystic ovary syndrome, serum copper

## Abstract

The association between serum copper and polycystic ovary syndrome (PCOS) lacks definitive conclusions, and the intricate interactions with in vitro fertilization (IVF) cycle characteristics in infertility remain insufficiently explored. This retrospective study included 560 patients with tubal infertility (no‐PCOS) and 266 patients with PCOS undergoing IVF at the Affiliated Suzhou Hospital of Nanjing Medical University from January 2018 to December 2022. Patients' basic characteristics, hormonal and metabolic parameters, essential trace elements, and IVF cycle characteristics were measured and analyzed. The results revealed a significantly elevated serum copper level in the PCOS group compared to the control group [17.27 (15.54, 19.67) vs 15.4 (13.87, 17.35), μmol/L; *p* < .001]. Spearman correlation analyses revealed a significant positive correlation between serum copper concentration and body mass index (BMI), fasting glucose (FG), triglyceride (TG), total cholesterol (TC), and low‐density lipoprotein (LDL) in the no‐PCOS group. Additionally, a notable negative correlation with high‐density lipoprotein (HDL) was observed (*r* = −.184, *p* < .001). Within the PCOS group, serum copper concentration correlated significantly with BMI (*r* = .198, *p* = .004) and TG (*r* = .214, *p* = .002). The linear trend analysis indicated no significant relationship between serum copper concentration and ovarian response as well as preimplantation outcomes in both groups after adjusting for confounding factors. Our study provided evidence of elevated serum copper concentration in PCOS patients, closely associated with lipid metabolism but showing no correlation with IVF outcomes. These findings provide valuable real‐world data, enriching our nuanced understanding of the role of copper in female fertility.

## Graphical abstract

This study investigates the relationship between serum copper and PCOS, exploring intricate interactions with IVF cycle characteristics in infertility. The lack of definitive conclusions in this area necessitates a thorough examination. The retrospective study, involving 266 PCOS patients and 560 controls undergoing IVF, revealed elevated serum copper levels in the PCOS group with associations to metabolic parameters but no significant correlation with IVF outcomes.

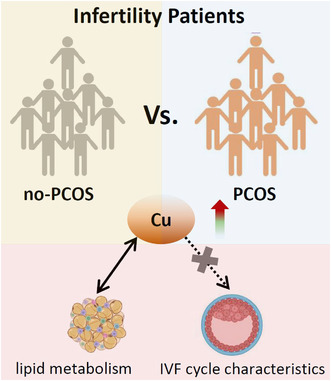

## 1 INTRODUCTION

The recent reports indicated that approximately 15% of the global reproductive‐age population suffers from infertility (Jiao et al., 2021). Notably, Polycystic Ovary Syndrome (PCOS) emerges as one of the predominant factors in female infertility, accounting for about 70% of anovulatory infertility (Bourgneuf et al., 2021). As a common endocrine disorder, PCOS affects 8%–13% of women in their reproductive years (Bordewijk et al., 2020), and 6%–18% of adolescent girls (Pena et al., 2020), marked by symptoms such as irregular menstruation, hyperandrogenism, and polycystic ovarian morphology. Additionally, PCOS is linked to metabolic disorders, such as dyslipidemia, insulin resistance, metabolic syndrome, and obesity, all highly intertwined with energy metabolism (Joham et al., 2022; Moghetti & Tosi, 2021).

There has been increasing recognition of the potential impact of trace elements on the pathophysiological mechanisms involved in PCOS. Trace elements, including copper, zinc, iron, and magnesium, are integral components of various proteins and metalloenzymes critical to cellular metabolic systems and oxidative stress pathways (Gonzalez‐Dominguez et al., 2023; Mohammadifard et al., 2019). Numerous studies have analyzed the distribution patterns of these elements in PCOS patients. For instance, multiple studies have revealed significantly lower circulating zinc levels in women with PCOS compared to healthy controls (Abedini et al., 2019; Ghanati et al., 2023). Another review found a potential decrease in serum magnesium concentration in overweight or obese women with PCOS (Babapour et al., 2021). A case–control study indicated elevated phosphorus levels in women with PCOS (Mahmoudi et al., 2010). Additionally, a recent systematic review and meta‐analysis reported higher serum iron concentrations in women with PCOS (Sharma et al., 2022). However, controversy remains regarding serum copper levels in PCOS.

Copper is a vital trace element for the human health, serving as a cofactor for various enzymes that play essential roles in vital cellular functions like energy production and antioxidant defense (Yang et al., 2024). Since the body cannot synthesize copper internally, it requires external intake through food. The estimated copper content in the human body is approximately 50 to 120 milligrams (Festa & Thiele, 2011; Kim et al., 2008). Maintaining proper copper homeostasis is crucial for overall physiological function, and imbalances are associated with various health conditions. Excessive copper accumulation can lead to cellular toxicity, and in extreme cases, cell death (Tardito et al., 2011). Driven by the biological magnification effect, the accumulation of copper in the body may result in a range of health issues, including potential impacts on reproductive health (Roychoudhury et al., 2016).

Kurdoglu et al. explored the association between copper levels and PCOS, revealing elevated copper concentrations in women with PCOS compared to the control participants (Kurdoglu et al., 2012). This finding sparked interest in understanding the potential impact of increased copper levels on infertility associated with PCOS. Subsequent reports also highlighted elevated serum copper levels in PCOS patients (Chakraborty et al., 2013; Kanafchian et al., 2020; Sun et al., 2019). However, Kirmizi et al. reported contrasting results, indicating lower copper concentrations in contrast to the control participants within the PCOS cohort (Kirmizi et al., 2020). Additionally, there is significant attention to the impact of copper on reproductive functions (Roychoudhury et al., 2016). A recent study indicated that elevated copper intake disrupts gut microbiota balance, induces oxidative stress and inflammatory response, interferes with hormone signal transduction, and influences the development of host follicles (Wang et al., 2023). Totally, the link between PCOS and copper has been studied for many years but has yet to yield definitive conclusions. Additionally, the intricate interactions between copper ions, hormonal imbalances, metabolic parameters, ovarian stimulation response in infertility patients, and IVF outcomes in the context of PCOS and infertility remain insufficiently explored.

Against this backdrop, our study aimed to explore the intricate interplay between the serum trace elements, hormonal distributions, and metabolic states in infertile patients with PCOS. Additionally, the study delved into the influence of trace element levels on ovarian response and preimplantation outcomes in these patients.

## 2 EXPERIMENTAL SECTION

### 2.1 Participants

This retrospective study enrolled 766 Chinese women diagnosed with infertility, aged ≤38 years. The participants comprised 266 individuals with PCOS and 560 individuals in the control group with tubal infertility (no‐PCOS). The diagnosis of PCOS relied on the modified Rotterdam Criteria requires fulfilling a minimum of two out of three criteria: (1) oligo‐ovulation/anovulation; (2) biochemical and/or clinical hyperandrogenemia, and the exclusion of potential causes of hyperandrogenemia; and (3) the ultrasound observation of polycystic ovarian morphology, characterized by the existence of 12 or more small follicles (2–9 mm) in each ovary or the ovarian volume exceeding 10 mL. Participants were excluded if they exhibited endometriosis, hyperprolactinemia, hypogonadotropic hypogonadism, thyroid dysfunction, premature ovarian failure, a history of recurrent abortions, chromosomal abnormalities, history of ovarian surgeries, or if their partners had severe teratozoospermia. Regular use of medications affecting hormone or lipid metabolism and the presence of clinically evident acute or chronic diseases, including infections or tumors, were also grounds for exclusion.

**FIGURE 1:**
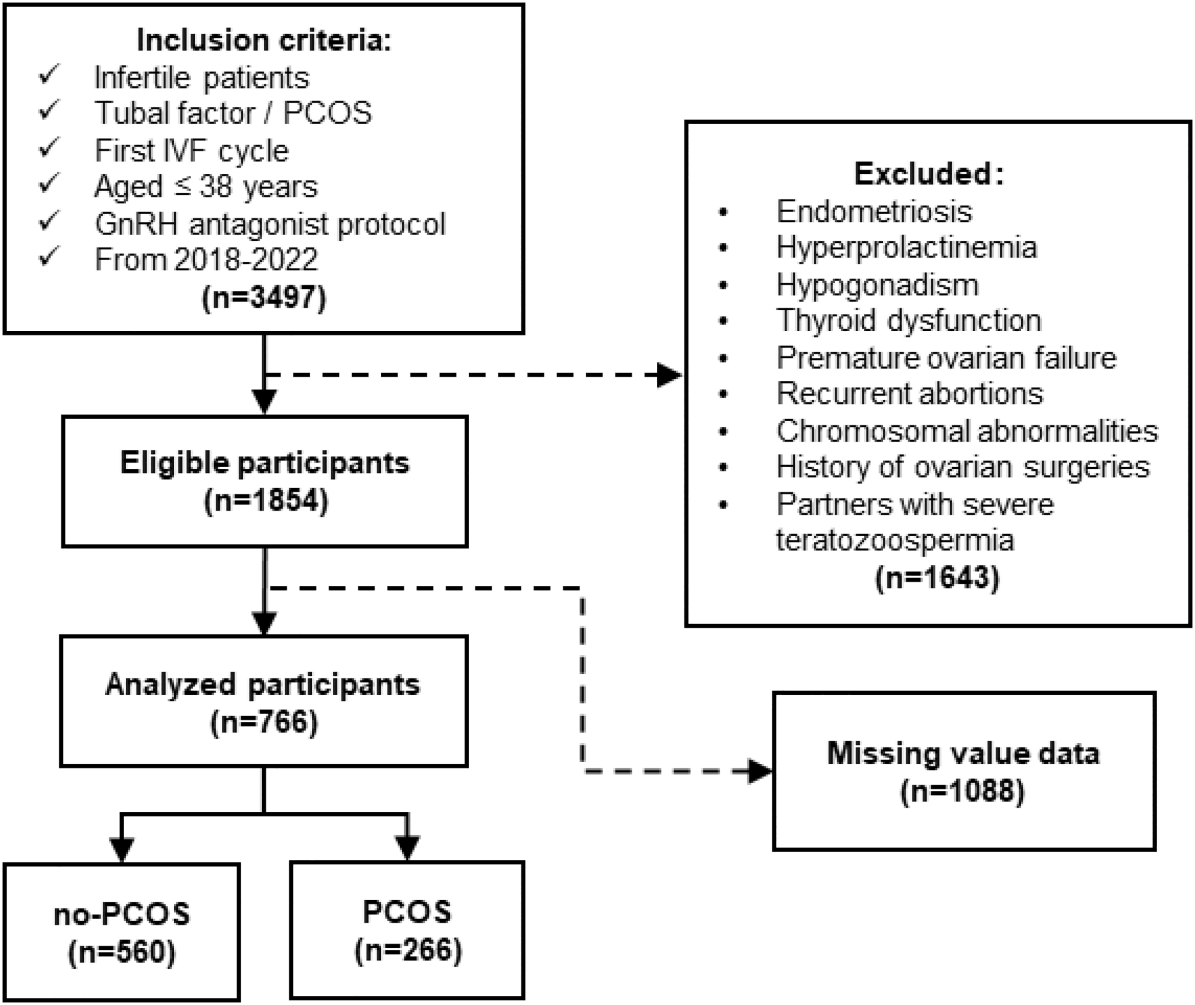
Flowchart of the participants in the study. PCOS, polycystic ovary syndrome; GnRH, gonadotropin‐releasing hormone.

All participants, drawn from the Center of Reproduction and Genetics (Suzhou Municipal Hospital, Suzhou, China), underwent the first IVF cycle from January 2018 to December 2022. Ovarian stimulation was conducted utilizing the GnRH antagonist protocol. The recruitment flow of the participants is shown in Figure 1. Detailed clinical data were retrieved from the system of Clinical Reproductive Medicine Management. The research followed the principles of the Helsinki Declaration and received approval from the Reproductive Medicine Ethics Committee (Suzhou Municipal Hospital). All participants provided informed consent.

### 2.2 Biochemical and hormone assays

Participants fasted overnight before blood sample collection, and the serum was stored at −80°C for subsequent analysis of biochemical and trace element concentrations. Serum biochemical parameters, including fasting glucose (FG), triglycerides (TG), high‐density lipoprotein (HDL), total cholesterol (TC), and low‐density lipoprotein (LDL), were assessed via the automated blood biochemical analyzer.

Serum concentrations of trace elements, including copper, iron, zinc, phosphorus, and magnesium, were assessed. Briefly, specific assay kits for each element were utilized, following the provided experimental protocols. For copper determination, the Quick Auto Neo Cu kit (SHINO‐TEST CORPORATION, Japan) was employed. Initially, a deproteinizing agent was applied to release serum copper ions bound to proteins (ceruloplasmin). Subsequently, the copper ions, reduced by ascorbic acid, reacted with a predetermined chelating agent, 4‐(3,5‐dibromo‐2‐pyridylazo)‐*N*‐ethyl‐*N*‐(3‐sulfopropyl) aniline derivative. The absorbance was then measured via comprehensive biochemical analysis to calculate the final concentration. Iron measurement was conducted using the Quick Auto Neo Fe kit (SHINO‐TEST CORPORATION, Japan). This kit utilizes a highly sensitive chelating agent (Nitroso‐PSAP) to quantify iron in serum. Iron (Fe3+) in the sample binds to transferrin, a globulin. Under acidic conditions, Fe3+ dissociates from transferrin. Subsequently, Fe3+ is reduced to Fe2+ by a reducing agent (ascorbic acid). Fe3+ forms a chelate compound with Nitroso‐PSAP, resulting in color formation. Zinc determination was performed using the Zn Assay Kit (Metallogenic Co., Ltd., Japan). Free and protein‐bound zinc ions react with 5‐Br‐PAPS to form a chelate complex, detected by measuring absorbance at 560 nm. Magnesium assessment was conducted using the Mg‐HR II Kit (FUJIFILM, Japan). Under alkaline conditions, magnesium forms a reddish‐purple, water‐soluble chelate complex with Xylenol Blue I (XB‐I), showing maximum absorption at 520 nm, while causing a decrease in absorbance at 620 nm (maximum absorption) for XB‐I. The magnesium concentration in the sample was determined by measuring the decrease in absorbance of Xylenol Blue I. Phosphorus determination utilized the P‐HR II assay kit (FUJIFILM, Japan). The serum sample was mixed with Color Solution A and Developer Solution B. Inorganic phosphorus in the sample reacted with molybdate in Developer Solution B to form phosphomolybdic acid, measured by absorbance.

Hormonal levels were assessed utilizing an automated electro‐chemiluminescent immunoassay system (Roche Diagnostics). Blood samples obtained during the initial follicular phase (7 am to 9 am) served as baseline of hormones, including follicle‐stimulating hormone (FSH), luteinizing hormone (LH), estradiol (E2), progesterone (P), anti‐Mullerian hormone (AMH), testosterone (T), and prolactin (PRL). On the trigger day, blood samples obtained (7 am to 9 am) were employed to measure trigger day levels of E2, LH, and P.

### 2.3 IVF procedure and definition

All participants were subjected to controlled ovarian hyperstimulation (COH) employing a protocol with GnRH antagonists. Ovulation was triggered when a minimum of three follicles were ≥18 mm or four follicles were ≥17 mm. The trigger involved the administration of GnRH agonist and/or human chorionic gonadotropin (hCG). Within 36 h, oocyte retrieval under ultrasound guidance was conducted. The standardized IVF protocol was then applied uniformly to all participants. The rate of oocyte recovery was calculated as the ratio of recovered oocytes to punctured follicles. The rate of MII oocyte was determined by dividing the number of metaphase II (MII) oocytes by the total retrieved oocytes. The rate of normal fertilization was determined by the number of two pronuclear zygotes divided by the inseminated oocytes. The rate of blastocyst formation was defined as the number of blastocysts formed divided by the normal fertilization oocytes. The rate of high‐quality embryo was determined by dividing the number of high‐quality embryos by the normal fertilization oocytes.

### 2.4 Statistical analyses

Analysis was carried out using Statistical Package for the Social Sciences version 27 (SPSS 27), and significance was set at *p* < .05 for two‐sided tests. Data normality was assessed through the Shapiro–Wilk normality test. Continuous variables between groups were compared using the nonparametric Mann–Whitney *U* test. The Spearman correlation analysis was employed to assess the correlation between parameters. Considering the distribution of the study cohort, serum copper levels were divided into four quartiles. Linear regression was employed to conduct a linear trend test for the median serum copper levels within each quartile. GraphPad Software (Prism 8.0, USA) was used to visualize the data.

## 3 RESULTS

### 3.1 Basic characteristics of no‐PCOS and PCOS participants

**FIGURE 2:**
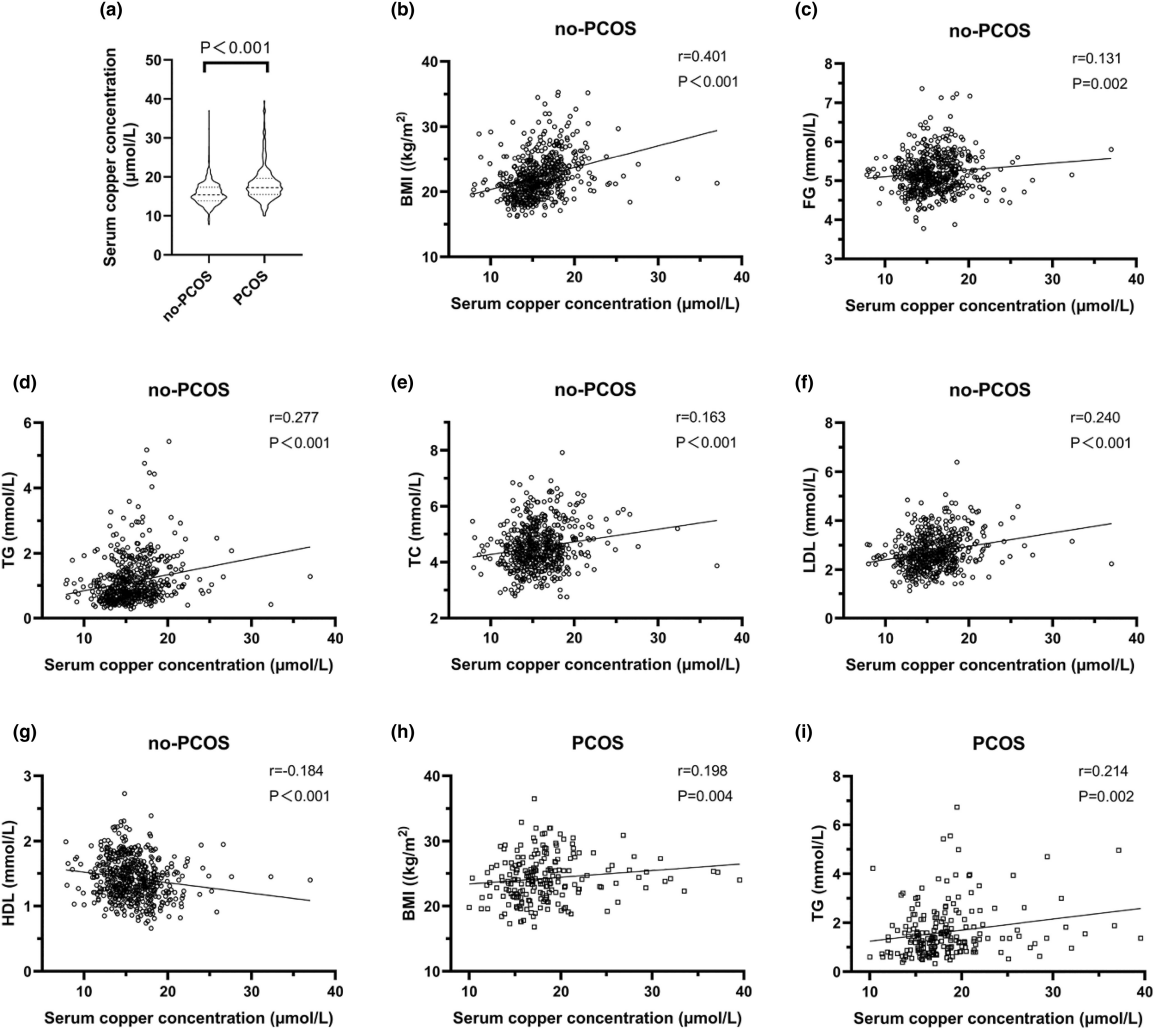
Visualization of serum copper concentration and its correlation with metabolic parameters. (a) serum copper concentration in the no‐PCOS and PCOS groups. (b–g) Scatter plot trend of serum copper concentrations with BMI, FG, TG, TC, LDL, and HDL in the no‐PCOS group. (h, i) Scatter plot trend of serum copper concentrations with BMI and TG in the PCOS group. BMI, body mass index; FG, fasting glucose; HDL, high‐density lipoprotein; LDL, low‐density lipoprotein; PCOS, polycystic ovary syndrome; TC, total cholesterol; TG, triglyceride.

Among 766 participants with infertility, 560 had solely tubal infertility (no‐PCOS), while 206 were diagnosed with PCOS. The clinical and biochemical characteristics of participants are outlined in Table 1. The PCOS group exhibited a significantly younger age compared to the no‐PCOS group [29 (27, 31) vs 31 (28, 33), years; *p* < .001]. Additionally, the duration of infertility was significantly shorter in the PCOS group compared to the no‐PCOS group (*p* = .022). In comparison to the no‐PCOS group, the PCOS group exhibited higher levels of BMI (*p* < .001), AMH (*p* < .001), and AFC (*p* < .001). Regarding baseline hormones, the PCOS participants exhibited elevated levels of LH (*p* < .001), LH/FSH (*p* < .001), and T (*p* < .001) but lower levels of FSH (*p* < .001) and PRL (*p* < .001). In terms of lipid metabolites, the PCOS participants demonstrated higher levels of TC (*p* < .001), TG (*p* < .001), and LDL (*p* < .001) but lower levels of HDL (*p* < .001). Furthermore, we conducted a comparison of trace element levels between the two groups, revealing elevated copper levels in the PCOS group [17.27 (15.54, 19.67) vs 15.4 (13.87, 17.35), PCOS vs no‐PCOS, μmol/L; *p* < .001] and Fe (*p* = .023). The disparity in copper levels between the two groups is depicted in Figure 2a.

**TABLE 1:** Basic characteristics and trace elements of no‐PCOS and PCOS groups [Median (P25, P75)].

Variables	no‐PCOS *N* = 560	PCOS *N* = 206	*Z*	*p*
Age (years)	31 (28, 33)	29 (27, 31)	−5.552	**<.001**
Infertile Duration (years)	3 (2, 5)	3 (2, 5)	−2.284	**.022**
BMI (kg/m^2^)	21.8 (20, 24)	24 (21.8, 26.4)	−7.395	**<.001**
AMH (μg/L)	3.76 (2.47, 5.83)	9.04 (6.7, 13.26)	−15.87	**<.001**
AFC (*n*)	16 (12, 20)	28 (22, 34.5)	−15.899	**<.001**
Baseline hormones				
FSH (mIU/mL)	7.45 (6.34, 8.68)	6.55 (5.63, 7.55)	−6.215	**<.001**
LH (mIU/mL)	4.22 (3.17, 5.64)	8.07 (4.93, 11.74)	−11.795	**<.001**
LH/FSH	0.58 (0.42, 0.78)	1.15 (0.79, 1.74)	−14.16	**<.001**
E2 (pg/mL)	36.2 (28.2, 49)	37 (28, 49.5)	−0.091	.928
P (ng/mL)	0.59 (0.39, 0.9)	0.61 (0.4, 0.93)	−0.326	.745
PRL (ng/mL)	15.42 (11.72, 21.19)	13.34 (9.83, 18.92)	−3.838	**<.001**
T (ng/mL)	0.47 (0.35, 0.59)	0.65 (0.49, 0.83)	−9.839	**<.001**
Glucolipid metabolites				
FG (mmol/L)	5.16 (4.88, 5.48)	5.21 (4.92, 5.59)	−1.609	.108
TG (mmol/L)	0.92 (0.69, 1.37)	1.36 (0.88, 1.93)	−7.11	**<.001**
TC (mmol/L)	4.47 (4.01, 4.98)	4.82 (4.24, 5.5)	−4.912	**<.001**
HDL (mmol/L)	1.41 (1.21, 1.63)	1.25 (1.09, 1.46)	−5.714	**<.001**
LDL (mmol/L)	2.6 (2.28, 3.14)	3.06 (2.57, 3.63)	−6.673	**<.001**
Trace elements				
Cu (μmol/L)	15.4 (13.87, 17.35)	17.27 (15.54, 19.67)	−8.065	**<.001**
Zn (μmol/L)	15.15 (13.7, 16.8)	14.9 (13.35, 17)	−0.347	.729
Fe (μmol/L)	15 (11.3, 19.9)	16.4 (12.45, 20.95)	−2.274	**.023**
P (mmol/L)	1.17 (1.06, 1.28)	1.19 (1.1, 1.29)	−1.8	.072
Mg (mmol/L)	0.91 (0.86, 0.95)	0.92 (0.87, 0.96)	−1.638	.101

Abbreviations: AFC, antral follicle count; AMH, anti‐mullerian hormone; BMI, body mass index; Cu, copper; E2, estradiol; Fe, iron; FG, fasting glucose; FSH, follicle‐stimulating hormone; HDL, high‐density lipoprotein; LDL, low‐density lipoprotein; LH, luteinizing hormone; Mg, magnesium; P, phosphorus; P, progesterone; PRL, prolactin; T, testosterone; TC, total cholesterol; TG, triglyceride; Zn, zinc.

Bold values were statistically significant.

### 3.2 IVF cycle characteristics of no‐PCOS and PCOS participants

Table 2 provides a summary of the ovarian response and preimplantation outcomes of the participants. The total gonadotropins (Gn) dose and total Gn treatment days were notably elevated in the PCOS group compared to the no‐PCOS group. Additionally, on the trigger day, the PCOS group showed a significant elevation in LH and E2 levels (*p* < .001). In line with expectations, the PCOS group showed a significant increase in retrieved oocytes, 2PN oocytes, MII oocytes, blastocysts, and high‐quality embryos compared to the no‐PCOS group. Regarding rates, the PCOS participants demonstrated significantly lower oocyte recovery rate [71.43 (61.49, 82.98) vs 79.17 (66.67, 90), PCOS vs no‐PCOS, %; *p* < .001], MII oocyte rate (*p* = .042), and normal fertilization rate (*p* = .005) compared to the no‐PCOS participants. However, there were no significant differences in blastocyst formation rate and high‐quality embryo rate between the two groups.

**TABLE 2:** IVF cycle characteristics of no‐PCOS and PCOS groups [Median (P25, P75)].

Variables	no‐PCOS *N* = 560	PCOS *N* = 206	*Z*	*p*
Total Gn dose (IU)	1543.75 (1200, 2000)	1400 (1143.75, 1800)	−3.415	**.001**
Total Gn treatment days	9 (8, 9)	9 (8, 10)	−4.184	**<.001**
Hormones on trigger day				
LH (mIU/mL)	2.48 (1.57, 4.09)	3.94 (2.19, 6.08)	−7.412	**<.001**
E2 (pg/mL)	2414 (1628.5, 3617.25)	3994 (2536.75, 5403)	−9.136	**<.001**
P (ng/mL)	0.73 (0.49, 1.04)	0.68 (0.43, 1.02)	−1.216	.224
Numbers (*n*)				
Retrieved oocytes	11 (8, 15)	17 (12, 24)	−9.397	**<.001**
MII oocytes	9 (7, 13)	15 (10, 20)	−8.657	**<.001**
2PN oocytes	6 (4, 9)	9 (6, 15)	−6.257	**<.001**
Blastocysts	3 (1, 5)	4 (2, 8)	−5.147	**<.001**
High‐quality embryos	4 (2, 6)	5 (3, 9)	−5.19	**<.001**
Rate (%)				
Oocyte recovery rate	79.17 (66.67, 90)	71.43 (61.49, 82.98)	−4.747	**<.001**
MII oocyte rate	88.12 (78.57, 100)	86.67 (75, 95.03)	−2.037	**.042**
Normal fertilization rate	80 (65.2, 92.86)	77.78 (57.14, 89.04)	−2.784	**.005**
Blastocyst formation rate	42.86 (20, 63.64)	50 (27.82, 66.67)	−1.664	.096
High‐quality embryo rate	66.67 (40, 83.33)	62.5 (44.05, 83.33)	−0.318	.751

Abbreviations: E2, estradiol; Gn, gonadotropins; LH, luteinizing hormone; M II, metaphase II; P, progesterone; 2PN, double pronuclei.

Bold values were statistically significant.

### 3.3 Correlation of serum copper concentration and baseline hormones and metabolic parameters

To explore the correlation between serum copper concentration and baseline hormones, as well as metabolic parameters, we conducted Spearman correlation analyses separately for the no‐PCOS participants and the PCOS participants. The study found no significant correlations between copper concentration and baseline hormones in both groups, while significant positive correlations were noted between copper and BMI (no‐PCOS: *r* = .401, *p* < .001; PCOS: *r* = .198, *p* = .004) in each group (Table 3). In the no‐PCOS group, serum copper concentration showed significant positive correlations with FG (*r* = .131, *p* = .002), TG (*r* = .277, *p* < .001), TC (*r* = .163, *p* < .001), and LDL (*r* = .240, *p* < .001), and a significant negative correlation with HDL (*r* = −.184, *p* < .001) (Table 3). In the PCOS group, only a significant positive correlation was observed between copper concentration and TG (*r* = .214, *p* = .002), with insignificant correlations with FG, TG, HDL, and LDL (Table 3). Additionally, scatter plots were generated to visualize the trends of parameters related to copper concentration (Figure 2b–i).

**TABLE 3:** Correlation of serum copper concentration and baseline hormones and metabolic parameters.

Variables	no‐PCOS		PCOS	
	*r*	*p*	*r*	*p*
FSH	−0.071	.093	−0.024	.728
LH	−0.026	.544	−0.028	.694
LH/FSH	0.016	.714	−0.017	.804
E2	−0.054	.204	0.029	.674
P	0.006	.885	0.013	.849
PRL	−0.038	.371	0.004	.954
T	0.078	.066	0.071	.094
AMH	−0.014	.738	−0.109	.12
BMI	**0.401**	**<.001**	**0.198**	**.004**
FG	**0.131**	**.002**	0.047	.502
TG	**0.277**	**<.001**	**0.214**	**.002**
TC	**0.163**	**<.001**	0.133	.057
HDL	**−0.184**	**<.001**	−0.005	.943
LDL	**0.240**	**<.001**	0.089	.203

*Note*: Spearman correlation analyses.

Bold values were statistically significant.

The correlation analysis between serum zinc, iron, magnesium, and phosphorus and baseline hormones and metabolic parameters revealed the following results (Tables S1–S4): In the non‐PCOS group, serum iron showed a positive correlation with TC (*r* = .099, *p* = .02), while in the PCOS group, iron exhibited a negative correlation with progesterone (*r* = −.16, *p* = .022). Serum zinc displayed a negative correlation with phosphorus in the non‐PCOS group (*r* = −.104, *p* = .014). Serum magnesium exhibited a negative correlation with E2 in the non‐PCOS group (*r* = −.086, *p* = .043), whereas in the PCOS group, it showed a negative correlation with FG (*r* = −.084, *p* = .046). Serum phosphorus demonstrated a positive correlation with FSH in the non‐PCOS group (*r* = −.109, *p* = .01).

### 3.4 Influences of serum copper levels on IVF outcomes

To evaluate the influence of serum copper levels on IVF outcomes, we initially categorized serum copper levels into quartiles. Subsequently, a linear trend was examined using the medians of serum copper level quartiles. The odds ratios (95% confidence intervals) for various parameters of IVF outcomes, stratified by serum copper concentration, are presented in Table 4. In the no‐PCOS group, serum copper levels showed statistically significant positive associations with total Gn dose (*p*‐trend < 0.001). In the PCOS group, serum copper levels exhibited significant positive correlations with parameters of total Gn dose (*p*‐trend = .005), retrieved oocytes (*p*‐trend = .03), and MII oocytes (*p*‐trend = .035). However, after adjusting for additional covariates, the associations between serum copper in the no‐PCOS participants and the total Gn dose, as well as copper concentrations in the PCOS participants and parameters of total Gn dose, retrieved oocytes, and MII oocytes, no longer retained statistical significance. It is noteworthy that serum copper levels in both groups showed no significant correlations with other parameters of IVF outcomes, including 2PN oocytes, blastocysts, and high‐quality embryos. Additionally, serum copper levels in both groups showed no significant correlations with other IVF outcome parameters, including oocyte recovery rate, MII oocyte rate, normal fertilization rate, blastocyst formation rate, and high‐quality embryo rate. Similarly, the impact of serum iron levels on IVF outcomes was assessed (Table S5). Only in the PCOS group, serum iron ions showed a negative correlation with the oocyte recovery rate, without adjusting for covariates (*p*‐trend = .042).

**TABLE 4:** OR [95% (CI)] for ovarian response and preimplantation outcomes according to serum copper concentration.

Variables	no‐PCOS					PCOS				
Copper levels, quartiles (μmol/L)	Q1	Q2	Q3	Q4	*p*‐trend	Q1	Q2	Q3	Q4	*p*‐trend
	≤13.87	13.87–15.4	15.4–17.35	>17.35		≤15.48	15.48–17.24	17.24–19.66	>19.66	
Total Gn dose										
Crude	1.00 (Ref.)	82.65 (−54.46, 219.76)	251.6 (115.22, 387.97)	318.67 (182.06, 455.29)	**<.001**	1.00 (Ref.)	134.51 (−84.9, 353.92)	296.82 (77.41, 516.23)	302.94 (82.47, 523.41)	**.005**
Adjusted model	1.00 (Ref.)	15.68 (−105.84, 137.2)	84.06 (−39.11, 207.23)	56.92 (−71.86, 185.71)	.274	1.00 (Ref.)	105.94 (−73.05, 284.94)	91.07 (−91.78, 273.91)	178.84 (−2.79, 360.47)	.075
Retrieved oocytes										
Crude	1.00 (Ref.)	0.08 (−1.34, 1.49)	0.1 (−1.3, 1.51)	−0.57 (−1.98, 0.84)	.446	1.00 (Ref.)	0.47 (−2.75, 3.69)	2.64 (−0.65, 5.93)	3.24 (−0.01, 6.49)	**.03**
Adjusted model	1.00 (Ref.)	0.16 (−1.21, 1.52)	0.17 (−1.22, 1.55)	−0.73 (−2.19, 0.72)	.347	1.00 (Ref.)	0.42 (−2.79, 3.64)	2.35 (−1.01, 5.71)	3 (−0.29, 6.28)	.055
MII oocytes										
Crude	1.00 (Ref.)	0.02 (−1.32, 1.37)	0.12 (−1.19, 1.44)	−0.21 (−1.54, 1.12)	.793	1.00 (Ref.)	0.22 (−2.69, 3.13)	2.8 (−0.19, 5.79)	2.8 (−0.24, 5.84)	**.035**
Adjusted model	1.00 (Ref.)	0.22 (−1.09, 1.53)	0.31 (−0.99, 1.62)	−0.12 (−1.5, 1.27)	.907	1.00 (Ref.)	0.16 (−2.74, 3.07)	2.55 (−0.5, 5.61)	2.73 (−0.34, 5.79)	.05
2PN oocytes										
Crude	1.00 (Ref.)	−0.06 (−1.11, 0.99)	−0.02 (−1.06, 1.03)	−0.11 (−1.16, 0.94)	.863	1.00 (Ref.)	−0.47 (−2.91, 1.97)	1.34 (−1.15, 3.83)	1.24 (−1.23, 3.71)	.196
Adjusted model	1.00 (Ref.)	0.13 (−0.9, 1.16)	0.24 (−0.81, 1.29)	0.14 (−0.97, 1.24)	.78	1.00 (Ref.)	−0.45 (−2.9, 1.99)	1.32 (−1.23, 3.88)	1.2 (−1.3, 3.7)	.238
High‐quality embryos										
Crude	1.00 (Ref.)	−0.35 (−1.18, 0.47)	−0.19 (−1.01, 0.63)	−0.26 (−1.08, 0.57)	.652	1.00 (Ref.)	0.71 (−1.03, 2.44)	0.94 (−0.83, 2.72)	0.78 (−0.98, 2.54)	.413
Adjusted model	1.00 (Ref.)	−0.22 (−1.04, 0.6)	0.01 (−0.82, 0.84)	−0.03 (−0.9, 0.85)	.936	1.00 (Ref.)	0.68 (−1.05, 2.42)	0.9 (−0.92, 2.72)	0.75 (−1.03, 2.53)	.451
Blastocysts										
Crude	1.00 (Ref.)	−0.38 (−1.13, 0.38)	−0.2 (−0.95, 0.55)	−0.17 (−0.93, 0.58)	.779	1.00 (Ref.)	−0.23 (−1.84, 1.39)	0.45 (−1.19, 2.1)	1.04 (−0.59, 2.67)	.144
Adjusted model	1.00 (Ref.)	−0.25 (−0.99, 0.5)	−0.05 (−0.81, 0.71)	−0.04 (−0.83, 0.76)	.957	1.00 (Ref.)	−0.19 (−1.8, 1.43)	0.58 (−1.11, 2.27)	1.1 (−0.55, 2.75)	.138
Oocyte recovery rate										
Crude	1.00 (Ref.)	−0.71 (−5.17, 3.75)	0.53 (−3.9, 4.97)	−2.39 (−6.86, 2.07)	.389	1.00 (Ref.)	−1.59 (−7.88, 4.7)	0.62 (−5.79, 7.04)	0 (−6.34, 6.35)	.856
Adjusted model	1.00 (Ref.)	−0.8 (−5.28, 3.69)	0.83 (−3.72, 5.37)	−1.65 (−6.43, 3.13)	.644	1.00 (Ref.)	−1.63 (−7.88, 4.62)	1.86 (−4.67, 8.39)	0.75 (−5.64, 7.14)	.662
MII oocyte rate										
Crude	1.00 (Ref.)	−3.2 (−8.98, 2.58)	3.42 (−2.32, 9.17)	2.81 (−2.98, 8.6)	.116	1.00 (Ref.)	0.16 (−9.73, 10.06)	−0.38 (−10.48, 9.71)	−8.35 (−18.34, 1.64)	.086
Adjusted model	1.00 (Ref.)	−2.68 (−8.46, 3.1)	4.4 (−1.46, 10.26)	4.53 (−1.64, 10.69)	.058	1.00 (Ref.)	−0.35 (−10.13, 9.43)	−0.02 (−10.24, 10.21)	−7.43 (−17.43, 2.58)	.138
Normal fertilization rate										
Crude	1.00 (Ref.)	−3.32 (−9.52, 2.87)	−1.19 (−7.35, 4.97)	0.97 (−5.23, 7.17)	.602	1.00 (Ref.)	−1.39 (−11.19, 8.41)	1.88 (−8.12, 11.88)	0.2 (−9.7, 10.09)	.857
Adjusted model	1.00 (Ref.)	−1.99 (−8.06, 4.08)	0.24 (−5.92, 6.39)	3.1 (−3.37, 9.58)	.278	1.00 (Ref.)	−1.54 (−11.33, 8.26)	2.35 (−7.89, 12.59)	0.75 (−9.26, 10.77)	.776
High‐quality embryo rate										
Crude	1.00 (Ref.)	−3.47 (−14.09, 7.14)	−0.4 (−10.95, 10.16)	3.05 (−7.58, 13.68)	.468	1.00 (Ref.)	7.18 (−4.07, 18.43)	−3.14 (−14.62, 8.34)	1.84 (−9.52, 13.2)	.895
Adjusted model	1.00 (Ref.)	−3.44 (−14.13, 7.25)	−0.02 (−10.85, 10.82)	3.94 (−7.46, 15.33)	.417	1.00 (Ref.)	7.04 (−4.22, 18.3)	−3.6 (−15.38, 8.17)	1.67 (−9.84, 13.19)	.919
Blastocyst formation rate										
Crude	1.00 (Ref.)	−7.28 (−16.69, 2.13)	−4.81 (−14.17, 4.55)	1.7 (−7.73, 11.12)	.589	1.00 (Ref.)	5.95 (−5.07, 16.96)	−1.11 (−12.35, 10.13)	7.11 (−4.02, 18.23)	.345
Adjusted model	1.00 (Ref.)	−6.83 (−16.28, 2.63)	−4 (−13.59, 5.59)	2.75 (−7.34, 12.83)	.5	1.00 (Ref.)	6.21 (−4.8, 17.23)	−0.04 (−11.55, 11.48)	7.71 (−3.55, 18.98)	.278

*Note*: The linear trend was examined using the medians of serum copper concentration quartiles. Crude, unadjusted for confounders; Adjusted model, adjusted for age, BMI, duration of infertility, and type of infertility.

Bold values were statistically significant.

## 4 DISCUSSION

In this investigation, we explored the basic characteristics, lipid metabolites, and essential trace elements of 560 tubal infertility participants and 206 PCOS‐related infertility participants. Consistent with previous reports, marked distinctions were identified between the no‐PCOS and PCOS groups concerning variables such as age, BMI, AMH, AFC, infertile duration, FSH, LH, FSH/LH ratio, PRL, and T, as well as TG, TC, HDL, and LDL. Notably, our results revealed a substantial elevation in copper concentrations among PCOS individuals compared to the no‐PCOS individuals. Further correlation analysis revealed a noteworthy positive correlation between serum copper levels and BMI, as well as TG in both groups. However, our results suggested that, after adjusting for confounding factors (age, BMI, duration of infertility, and type of infertility), serum copper levels did not emerge as a significant influencing factor for IVF outcomes.

Copper, an essential trace nutrient crucial for human health, is primarily acquired through dietary intake and absorbed in the duodenum. With typical dietary intake levels ranging from 1 to 5 mg/day, absorption rates range from 12% to 60% (Henriksen & Arnesen, 2023), controlled by host conditions and dietary elements. Copper plays crucial roles in energy production, antioxidant defense, neurotransmitter production, and connective tissue formation by acting as a key cofactor for various enzymes participated in vital cellular processes (Tsang et al., 2021; Uriu‐Adams & Keen, 2005). Imbalances in copper levels are associated with the development of numerous chronic inflammatory conditions (Han, 2023).

As of now, limited research has explicitly investigated the link between serum copper levels and PCOS. Kurdoglu et al. examined the concentrations of various essential trace elements in the serum of 65 women, including 35 patients with PCOS and 30 controls. Their findings indicated a notable increase in serum copper levels among individuals with PCOS (Kurdoglu et al., 2012). Similarly, a study involving 1137 individuals reported that PCOS individuals showed significantly elevated copper levels compared to those without PCOS (Li et al., 2017). However, some studies have yielded conflicting conclusions. In a case–control study, it was reported no significant disparity in zinc and copper levels between PCOS patients and the control patients (Sharif et al., 2017). Additionally, Kirmizi et al. reported a substantial reduction in serum copper concentrations within the PCOS participants when compared to the control participants (*p* = .030) (Kirmizi et al., 2020). In light of these mixed findings, a conclusive interpretation remains elusive. Jiang et al. conducted a meta‐analysis in 2021, encompassing nine studies with a total of 1168 PCOS participants and 1106 controls. This meta‐analysis generally indicated an increase in copper concentrations in PCOS participants, and overall heterogeneity was unrelated to country subgroups. Subsequently, a 2022 meta‐analysis comprising 32 studies with 2317 PCOS cases and 1898 controls revealed significantly elevated serum concentrations of copper, cobalt, chromium, and iron in women with PCOS compared to the control group (Sharma et al., 2022). Here, our retrospective study revealed significantly elevated serum copper levels in the PCOS participants compared to the no‐PCOS participants [17.27 (15.54, 19.67) vs 15.4 (13.87, 17.35), μmol/L; *p* < .001]. Overall, existing evidence leans toward a significant elevation of serum copper concentrations in patients with PCOS.

PCOS is closely associated with metabolic disturbances, and patients with PCOS often exhibit abnormalities in glucose and lipid metabolism (Anagnostis et al., 2018). Previous studies have reported that lipid metabolism disorders in PCOS patients are characterized by elevated levels of LDL, TC, and TG, coupled with decreased levels of HDL (Xu et al., 2019). Our results were consistent with these observations. A latest study explored the relationship between serum trace elements and biochemical variables in PCOS patients, revealing associations between specific trace elements and HDL‐C, albumin, and cholesterol levels (Sharma et al., 2024). Similarly, the impact of copper on lipid metabolism has been extensively studied. Copper actively participates in diverse lipid metabolic processes, encompassing the synthesis of fatty acids, cholesterol, and lipoproteins. Variations in copper concentrations are likely linked to alterations in lipoprotein levels (Yang et al., 2024). It reported that in healthy individuals, serum copper levels positively correlate with lipid peroxidation, apolipoprotein B, triglycerides, and total cholesterol (Craig et al., 1995). A study found a direct correlation between elevated copper levels and increased concentrations of TC and HDL (Song et al., 2018). Additionally, higher copper levels were linked to an elevated risk of dyslipidemia, characterized by increased TC and LDL (Song et al., 2018). Our investigation indicated a notable positive correlation between copper concentrations and LDL, TG, and TC in the no‐PCOS participants. Interestingly, in the PCOS participants, a significant association was observed only with serum copper levels and TG. This may be attributed to the relatively higher levels of copper and lipid parameters in PCOS participants, leading to a weakening of their association.

As a redox‐active metal, copper can induce oxidative stress in cells when present in excess or deficiency (Li et al., 2023). Excess copper can induce oxidative stress via the Fenton reaction, resulting in the production of highly hydroxyl radicals (Hao et al., 2021). Another mechanism involves the depletion of antioxidants, including low‐molecular‐weight antioxidants like glutathione, alpha‐tocopherol, and ascorbic acid, along with antioxidant enzymes like glutathione peroxidase, superoxide dismutase, and catalase. These enzymes can bind to metal ions, inhibiting their catalytic activity and reducing the production of reactive oxygen species (ROS) (Jomova & Valko, 2011). Conversely, copper deficiency can compromise various components of the antioxidant defense system, including copper/zinc‐SOD and ceruloplasmin, and diminish the activities of enzymes such as catalase and selenium‐dependent glutathione peroxidase that do not contain copper, thereby altering other ROS scavengers such as metallothionein and glutathione (Uriu‐Adams & Keen, 2005).

The imbalance of copper has also been shown to adversely impact the reproductive system (Roychoudhury et al., 2016). Li et al. found that prolonged exposure to elevated dietary copper levels can alter gut microbiota composition, leading to inflammation, oxidative stress, and disruptions in hormone signaling, ultimately influencing ovarian follicle development (Wang et al., 2023). Evidence suggests that maintaining an optimal copper level in the ovarian is beneficial for the maturation and development of oocytes, potentially by influencing hormone secretion or directly improving oocyte quality (Peacey et al., 2020). Specifically, Ingle et al. reported increased oocyte recovery with urinary copper concentrations (Ingle et al., 2017), and Gonzalez‐Martin et al. found that copper levels in follicular fluid and plasma improved IVF cycle characteristics (Gonzalez‐Martin et al., 2023). It should be noted that the sample sizes for the aforementioned two studies were 58 and 60, respectively. Moreover, neither of the studies conducted a subgroup analysis of participants. Our study included 560 participants with tubal infertility and 206 participants with PCOS‐related infertility, assessing the impact of copper on reproductive outcomes in each group. The findings unveiled a significant correlation between copper levels and the total amount of Gn used in both groups. However, this association lost significance after adjusting for potential confounders including age, BMI, duration of infertility, and type of infertility. A similar pattern was observed in the PCOS participants concerning the association between copper levels and outcomes of retrieved oocytes and MII oocytes. Overall, our results indicated no significant correlation between copper levels and IVF cycle characteristics after controlling for confounding factors. The discrepancy with previous reports may be attributed to variations in the source of copper ions, sample size, classification of participant subgroups, and the control of confounding factors.

This study had several limitations. First, the single‐center recruitment of all participants may introduce center‐specific biases, limiting the generalizability of the findings. Second, being a retrospective study, inherent biases and confounding factors may be present, although efforts were made to adjust for potential confounders. Furthermore, the study solely obtained copper parameters in serum, and a more comprehensive analysis incorporating copper levels in plasma, follicular fluid, and urine would provide a more holistic understanding. Lastly, the cross‐sectional design impedes causal inference, underscoring the necessity for future longitudinal studies to elucidate how copper levels longitudinally impact reproductive outcomes.

## 5 CONCLUSIONS

Our study indicated a significant elevation in serum copper levels in infertility patients with PCOS. Additionally, a strong association between serum copper and lipid metabolism, particularly triglycerides, was observed. In terms of IVF outcomes, there was no discernible correlation between serum copper and oocyte quantity or associated rates. This suggests that copper is a valuable indicator reflecting the metabolic status of infertility patients, particularly lipid metabolism. Further exploration of the potential interaction mechanisms between copper and lipid metabolism will enhance our understanding of the metabolic abnormalities in PCOS. The limitations of this study underscored the necessity for future research, particularly prospective longitudinal studies incorporating diverse types of copper parameters and conducting larger, more representative cohorts. In summary, this study provided valuable insights, offering real‐world data to enhance the insight into the role of copper in female fertility.

## AUTHOR CONTRIBUTIONS

**Yanping Liu:** Conceptualization (equal); investigation (equal); methodology (equal); project administration (equal); writing – original draft (equal). **Wei Zhang:** Investigation (equal); methodology (equal); writing – original draft (equal). **Zhenxing Liu:** Funding acquisition (equal); resources (equal); software (equal). **Aiyan Zheng:** Methodology (equal); resources (equal). **Baoquan Liang:** Funding acquisition (equal); resources (equal); software (equal). **Hong Li:** Conceptualization (equal); funding acquisition (equal); supervision (equal); writing – review and editing (equal). **Qingxia Meng:** Conceptualization (equal); funding acquisition (equal); project administration (equal); supervision (equal); writing – review and editing (equal).

## FUNDING INFORMATION

This research was funded by The National Key Research and Development Program of China (2022YFC2702901), Suzhou key clinical diseases funding (LCZX202109), Suzhou Youth Project of Science and Education for Medicine (KJXW2022036), and Suzhou Science and Technology Plan Project (SKY2021056).

## CONFLICT OF INTEREST STATEMENT

The authors declare no conflict of interest.

## Supporting information


**Table S1.** Correlation of serum iron concentration with baseline hormones and metabolic parameters.
**TABLE S2.** Correlation of serum zinc concentration with baseline hormones and metabolic parameters.
**TABLE S3.** Correlation of serum magnesium concentration with baseline hormones and metabolic parameters.
**TABLE S4.** Correlation of serum phosphorus concentration with baseline hormones and metabolic parameters.
**TABLE S5.** OR [95% (CI)] for ovarian response and preimplantation outcomes according to serum iron concentration.

## Data Availability

Data are available upon request.
