# Supplementary material for: Serum copper assessment in patients with polycystic ovary syndrome and tubal infertility: A retrospective 5‐year study
Source: Food Sci Nutr. 2024 Jun 10;12(8):5979–89. doi: 10.1002/fsn3.4258 (PMC11317741; doi:10.1002/fsn3.4258)
Supplement: Supplementary file 1 — Table S1. Correlation of serum iron concentration with baseline hormones and metabolic parameters. TABLE S2. Correlation of serum zinc concentration with baseline hormones and metabolic parameters. TABLE S3. Correlation of serum magnesium concentration with baseline hormones and metabolic parameters. TABLE S4. Correlation of serum phosphorus concentration with baseline hormones and metabolic parameters. TABLE S5. OR [95% (CI)] for ovarian response and preimplantation outcomes according to serum iron concentration. [file FSN3-12-5979-s001.docx]

**Table S1.** Correlation of serum iron concentration with baseline hormones and metabolic parameters

| Variables | NO-PCOS | |  | PCOS | |
| --- | --- | --- | --- | --- | --- |
|  | r | P |  | r | P |
| FSH | -0.051 | 0.228 |  | 0.047 | 0.506 |
| LH | -0.049 | 0.251 |  | -0.014 | 0.836 |
| LH/FSH | 0.02 | 0.643 |  | -0.057 | 0.416 |
| E2 | 0.049 | 0.244 |  | 0.051 | 0.471 |
| P | 0.038 | 0.363 |  | **-0.160** | **0.022** |
| PRL | -0.052 | 0.218 |  | -0.08 | 0.251 |
| T | 0.07 | 0.099 |  | 0.093 | 0.186 |
| AMH | 0.015 | 0.718 |  | -0.02 | 0.781 |
| BMI | -0.015 | 0.717 |  | 0.069 | 0.322 |
| FG | -0.049 | 0.248 |  | -0.012 | 0.869 |
| TG | 0.078 | 0.065 |  | 0.063 | 0.367 |
| TC | **0.099** | **0.02** |  | 0.042 | 0.547 |
| HDL | 0.073 | 0.086 |  | 0.089 | 0.201 |
| LDL | 0.082 | 0.053 |  | 0.089 | 0.203 |

Spearman correlation analyses.

**Table S2.** Correlation of serum zinc concentration with baseline hormones and metabolic parameters

| Variables | NO-PCOS | |  | PCOS | |
| --- | --- | --- | --- | --- | --- |
|  | r | P |  | r | P |
| FSH | 0.061 | 0.146 |  | 0.016 | 0.822 |
| LH | -0.019 | 0.656 |  | 0.019 | 0.787 |
| LH/FSH | -0.077 | 0.068 |  | 0.014 | 0.841 |
| E2 | -0.011 | 0.793 |  | -0.029 | 0.682 |
| P | **-0.104** | **0.014** |  | 0.029 | 0.676 |
| PRL | 0.035 | 0.405 |  | 0.028 | 0.694 |
| T | -0.061 | 0.15 |  | -0.058 | 0.405 |
| AMH | -0.053 | 0.208 |  | 0.065 | 0.354 |
| BMI | 0.025 | 0.56 |  | 0.018 | 0.792 |
| FG | 0.057 | 0.178 |  | 0.086 | 0.218 |
| TG | 0.036 | 0.389 |  | 0.073 | 0.296 |
| TC | 0.049 | 0.245 |  | -0.082 | 0.241 |
| HDL | 0.05 | 0.236 |  | -0.091 | 0.192 |
| LDL | 0.035 | 0.405 |  | -0.062 | 0.376 |

Spearman correlation analyses.

**Table S3.** Correlation of serum magnesium concentration with baseline hormones and metabolic parameters

| Variables | NO-PCOS | |  | PCOS | |
| --- | --- | --- | --- | --- | --- |
|  | r | P |  | r | P |
| FSH | 0.071 | 0.092 |  | 0.045 | 0.525 |
| LH | 0.081 | 0.054 |  | -0.086 | 0.221 |
| LH/FSH | 0.022 | 0.597 |  | -0.098 | 0.162 |
| E2 | **-0.086** | **0.043** |  | -0.103 | 0.14 |
| P | 0.016 | 0.711 |  | 0.008 | 0.906 |
| PRL | 0.034 | 0.417 |  | 0.055 | 0.435 |
| T | 0.035 | 0.414 |  | -0.003 | 0.96 |
| AMH | 0.014 | 0.748 |  | 0.037 | 0.602 |
| BMI | -0.06 | 0.153 |  | -0.086 | 0.221 |
| FG | -0.076 | 0.073 |  | **-0.084** | **0.046** |
| TG | -0.004 | 0.93 |  | 0.051 | 0.463 |
| TC | 0.08 | 0.059 |  | -0.045 | 0.523 |
| HDL | 0.05 | 0.239 |  | -0.129 | 0.064 |
| LDL | 0.041 | 0.333 |  | 0.003 | 0.965 |

Spearman correlation analyses.

**Table S4.** Correlation of serum phosphorus concentration with baseline hormones and metabolic parameters

| Variables | NO-PCOS | |  | PCOS | |
| --- | --- | --- | --- | --- | --- |
|  | r | P |  | r | P |
| FSH | **0.109** | **0.01** |  | -0.023 | 0.742 |
| LH | 0.033 | 0.429 |  | -0.044 | 0.53 |
| LH/FSH | -0.044 | 0.304 |  | -0.032 | 0.651 |
| E2 | 0.054 | 0.205 |  | 0.001 | 0.988 |
| P | -0.002 | 0.964 |  | -0.034 | 0.625 |
| PRL | -0.054 | 0.208 |  | -0.124 | 0.075 |
| T | 0.011 | 0.803 |  | -0.01 | 0.881 |
| AMH | -0.045 | 0.29 |  | -0.091 | 0.192 |
| BMI | -0.061 | 0.154 |  | -0.023 | 0.746 |
| FG | -0.076 | 0.072 |  | -0.021 | 0.761 |
| TG | -0.066 | 0.118 |  | 0.047 | 0.501 |
| TC | 0.072 | 0.089 |  | -0.051 | 0.47 |
| HDL | 0.067 | 0.056 |  | -0.101 | 0.148 |
| LDL | 0.012 | 0.769 |  | -0.023 | 0.738 |

Spearman correlation analyses.

**Table S5.** OR [95% (CI)] for ovarian response and preimplantation outcomes according to serum iron concentration

| Variables | no-PCOS | | | | |  | PCOS | | | | |
| --- | --- | --- | --- | --- | --- | --- | --- | --- | --- | --- | --- |
| Copper levels,  quartiles (μmol/L) | Q1 | Q2 | Q3 | Q4 | p-trend |  | Q1 | Q2 | Q3 | Q4 | p-trend |
|  | ≤13.87 | 13.87 - 15.4 | 15.4 - 17.35 | ＞17.35 |  |  | ≤15.48 | 15.48 - 17.24 | 17.24 - 19.66 | ＞19.66 |  |
| Total Gn dose | | | | | | | | | | | |
| Crude | 1.00 (Ref.) | -49.08 (-188.76, 90.61) | 2.47 (-138.23, 143.17) | -62.78 (-202.72, 77.15) | 0.512 |  | 1.00 (Ref.) | -61.73 (-284.54, 161.08) | 128.29 (-95.60, 352.19) | 171.92 (-51.98, 395.82) | 0.058 |
| Adjusted model | 1.00 (Ref.) | -21.86 (-142.89, 99.16) | -2.84 (-124.49, 118.80) | -44.61 (-165.53, 76.31) | 0.531 |  | 1.00 (Ref.) | -60.94 (-242.75, 120.86) | 147.83 (-33.71, 329.38) | 74.55 (-108.59, 257.68) | 0.181 |
| Retrieved oocytes | | | | | | | | | | | |
| Crude | 1.00 (Ref.) | -1.20 (-2.61, 0.21) | -0.37 (-1.79, 1.05) | 0.02 (-1.39, 1.43) | 0.624 |  | 1.00 (Ref.) | -1.66 (-4.95, 1.62) | -0.64 (-3.93, 2.64) | -3.03 (-6.37, 0.30) | 0.115 |
| Adjusted model | 1.00 (Ref.) | -1.16 (-2.53, 0.20) | -0.26 (-1.63, 1.11) | -0.00 (-1.36, 1.35) | 0.619 |  | 1.00 (Ref.) | -1.51 (-4.83, 1.80) | -0.62 (-3.92, 2.68) | -3.20 (-6.56, 0.17) | 0.095 |
| MII oocytes | | | | | | | | | | | |
| Crude | 1.00 (Ref.) | -1.03 (-2.36, 0.30) | -0.23 (-1.57, 1.12) | -0.38 (-1.72, 0.96) | 0.919 |  | 1.00 (Ref.) | -0.97 (-4.03, 2.09) | 0.01 (-3.03, 3.05) | -1.25 (-4.36, 1.86) | 0.547 |
| Adjusted model | 1.00 (Ref.) | -1.07 (-2.37, 0.23) | -0.23 (-1.54, 1.08) | -0.47 (-1.78, 0.83) | 0.824 |  | 1.00 (Ref.) | -0.89 (-3.98, 2.19) | 0.00 (-3.06, 3.06) | -1.38 (-4.52, 1.75) | 0.487 |
| 2PN oocytes | | | | | | | | | | | |
| Crude | 1.00 (Ref.) | -1.36 (-2.62, -0.10) | -0.39 (-1.67, 0.88) | -0.48 (-1.74, 0.78) | 0.341 |  | 1.00 (Ref.) | -1.44 (-4.37, 1.49) | -0.75 (-3.69, 2.18) | -2.15 (-5.13, 0.83) | 0.263 |
| Adjusted model | 1.00 (Ref.) | -1.31 (-2.54, -0.08) | -0.29 (-1.53, 0.94) | -0.49 (-1.72, 0.73) | 0.346 |  | 1.00 (Ref.) | -1.36 (-4.32, 1.61) | -0.79 (-3.74, 2.16) | -2.32 (-5.33, 0.69) | 0.247 |
| High-quality embryos | | | | | | | | | | | |
| Crude | 1.00 (Ref.) | -0.63 (-1.46, 0.19) | 0.11 (-0.72, 0.94) | -0.28 (-1.10, 0.54) | 0.92 |  | 1.00 (Ref.) | -0.56 (-2.33, 1.20) | -0.82 (-2.58, 0.95) | -0.84 (-2.63, 0.95) | 0.345 |
| Adjusted model | 1.00 (Ref.) | -0.59 (-1.41, 0.22) | 0.16 (-0.66, 0.98) | -0.28 (-1.10, 0.53) | 0.901 |  | 1.00 (Ref.) | -0.49 (-2.28, 1.30) | -0.85 (-2.63, 0.94) | -0.86 (-2.68, 0.96) | 0.327 |
| Blastocysts | | | | | | | | | | | |
| Crude | 1.00 (Ref.) | -0.37 (-1.13, 0.38) | 0.28 (-0.48, 1.04) | 0.06 (-0.69, 0.81) | 0.515 |  | 1.00 (Ref.) | -0.25 (-1.89, 1.40) | -0.70 (-2.34, 0.95) | -0.46 (-2.13, 1.22) | 0.525 |
| Adjusted model | 1.00 (Ref.) | -0.32 (-1.06, 0.43) | 0.33 (-0.42, 1.08) | 0.06 (-0.68, 0.80) | 0.524 |  | 1.00 (Ref.) | -0.25 (-1.92, 1.42) | -0.71 (-2.38, 0.95) | -0.42 (-2.12, 1.28) | 0.551 |
| Oocyte recovery rate | | | | | | | | | | | |
| Crude | 1.00 (Ref.) | 0.92 (-3.54, 5.37) | -0.79 (-5.28, 3.69) | 3.72 (-0.72, 8.17) | 0.143 |  | 1.00 (Ref.) | -2.39 (-8.64, 3.86) | 1.27 (-4.98, 7.52) | -7.65 (-13.99, -1.30) | **0.043** |
| Adjusted model | 1.00 (Ref.) | 0.74 (-3.73, 5.20) | -0.94 (-5.43, 3.55) | 3.67 (-0.77, 8.12) | 0.145 |  | 1.00 (Ref.) | -2.14 (-8.47, 4.20) | 1.27 (-5.03, 7.57) | -7.16 (-13.60, -0.73) | 0.062 |
| MII oocyte rate | | | | | | | | | | | |
| Crude | 1.00 (Ref.) | 2.23 (-3.57, 8.04) | 1.02 (-4.83, 6.86) | -2.74 (-8.53, 3.06) | 0.257 |  | 1.00 (Ref.) | -0.14 (-10.24, 9.97) | 5.39 (-4.72, 15.49) | 0.74 (-9.52, 11.00) | 0.702 |
| Adjusted model | 1.00 (Ref.) | 2.47 (-3.33, 8.27) | 0.98 (-4.86, 6.81) | -2.69 (-8.46, 3.09) | 0.25 |  | 1.00 (Ref.) | 0.09 (-10.04, 10.21) | 4.84 (-5.23, 14.92) | 0.64 (-9.65, 10.93) | 0.737 |
| Normal fertilization rate | | | | | | | | | | | |
| Crude | 1.00 (Ref.) | 1.87 (-4.34, 8.09) | 2.10 (-4.16, 8.35) | 1.53 (-4.67, 7.73) | 0.666 |  | 1.00 (Ref.) | 0.31 (-9.63, 10.26) | -2.08 (-12.02, 7.87) | 0.78 (-9.32, 10.88) | 0.967 |
| Adjusted model | 1.00 (Ref.) | 2.62 (-3.46, 8.69) | 2.21 (-3.90, 8.32) | 1.68 (-4.37, 7.73) | 0.677 |  | 1.00 (Ref.) | 0.49 (-9.61, 10.59) | -2.37 (-12.41, 7.68) | 0.86 (-9.40, 11.12) | 0.978 |
| High-quality embryo rate | | | | | | | | | | | |
| Crude | 1.00 (Ref.) | -7.00 (-17.62, 3.63) | -3.50 (-14.21, 7.20) | -5.26 (-15.87, 5.35) | 0.493 |  | 1.00 (Ref.) | -5.74 (-17.13, 5.64) | -12.05 (-23.43, -0.66) | -3.73 (-15.28, 7.83) | 0.422 |
| Adjusted model | 1.00 (Ref.) | -6.96 (-17.63, 3.72) | -3.65 (-14.39, 7.08) | -5.24 (-15.87, 5.39) | 0.49 |  | 1.00 (Ref.) | -5.55 (-17.12, 6.02) | -12.29 (-23.80, -0.78) | -3.70 (-15.46, 8.05) | 0.411 |
| Blastocyst formation rate | | | | | | | | | | | |
| Crude | 1.00 (Ref.) | -8.02 (-17.46, 1.42) | -3.11 (-12.62, 6.40) | -3.37 (-12.79, 6.06) | 0.78 |  | 1.00 (Ref.) | -4.43 (-15.65, 6.80) | -5.44 (-16.67, 5.78) | 0.46 (-10.94, 11.85) | 0.929 |
| Adjusted model | 1.00 (Ref.) | -7.74 (-17.20, 1.73) | -3.02 (-12.54, 6.50) | -3.35 (-12.78, 6.07) | 0.77 |  | 1.00 (Ref.) | -4.62 (-16.00, 6.77) | -5.50 (-16.82, 5.83) | 1.00 (-10.58, 12.57) | 0.859 |

The linear trend was examined using the medians of serum iron concentration quartiles.

Crude, unadjusted for confounders; Adjusted model, adjusted for age, BMI, duration of infertility, and type of infertility.
